# Primary Splenic Epidermoid Cyst: A Case Report

**DOI:** 10.7759/cureus.22799

**Published:** 2022-03-03

**Authors:** TKeyah T Gray, Vijaykumar Patel, Megan Timpone, Kelsee Felux

**Affiliations:** 1 General Surgery, Ross University School of Medicine, Los Angeles, USA; 2 General Surgery, Wellstar Atlanta Medical Center, East Point, USA; 3 General Surgery, Ross University School of Medicine, Atlanta, USA

**Keywords:** minimally invasive surgery, percutaneous drainage, splenectomy, open versus laparoscopic, primary splenic epidermoid cyst

## Abstract

Primary splenic epidermal cysts, a type I splenic lesion, are very uncommon and usually found coincidentally. In this report, we present a primary splenic epidermal cyst that presented as a mass in the left upper quadrant associated with sharp pain, early satiety, and constipation. We review the classification of splenic cysts with a detailed look into the causes and types of type I cysts. We discuss the different treatment options, how current and past surgical options are controversial, and indications for splenectomy in spleen cysts. We explore how percutaneous drainage as a bridge to splenectomy may have been beneficial in a splenic cyst of great size. This splenic cyst was attempted laparoscopically but converted to an open splenectomy after complications. The patient recovered with no difficulties postoperatively.

## Introduction

Splenic cysts are rare, and they are commonly found incidentally. Splenic lesions are classified into Type I - primary (true) cysts with a histological finding containing an epithelial lining of parasitic or non-parasitic origin and Type II - secondary (false) cysts without an epithelial lining [1]. Of these splenic lesions, primary splenic epidermoid cysts constitute roughly 10% and are typically diagnosed in children and young adults [2]. Most cases are asymptomatic; however, as the cysts enlarge, patients generally present complaining of abdominal symptoms. The primary imaging modalities for diagnosis consist of abdominal ultrasound (US) and computerized tomography (CT), with the most accurate method being CT. Typically, treatment for splenic cysts is a splenectomy, but recent studies over the past decade or more show that conservative treatment of the spleen has reduced short and long-term complications. We are reporting a case of a 37-year-old female with a large splenic epidermoid cyst measuring 13 x 15 x 21 cm. Initially, we attempted removal via laparoscopic approach; however, due to its larger size, the decision was made to convert to an open total splenectomy.

## Case presentation

A 37-year-old African American female with no significant past medical history presented to the clinic complaining of a left upper quadrant mass that she discovered shortly after giving birth 11 months prior. The mass had been slowly enlarging since the delivery of her baby and was associated with sharp pain. The pain was exacerbated by walking and relieved by lying down. On further questioning, the patient attested to a concurrent history of early satiety and constipation. She denied rectal bleeding, hematochezia, nausea, and vomiting. The patient had no prior history of abdominal surgeries, pancreatitis, alcohol abuse, gallstone disease, or trauma to the abdomen.

Upon physical examination, visible asymmetry was noted in the left upper quadrant. A well-defined mass was palpated below the left costal margin. Abdominal tenderness in the left upper quadrant was not appreciated nor rebound tenderness or guarding on palpation.

**Figure 1:**
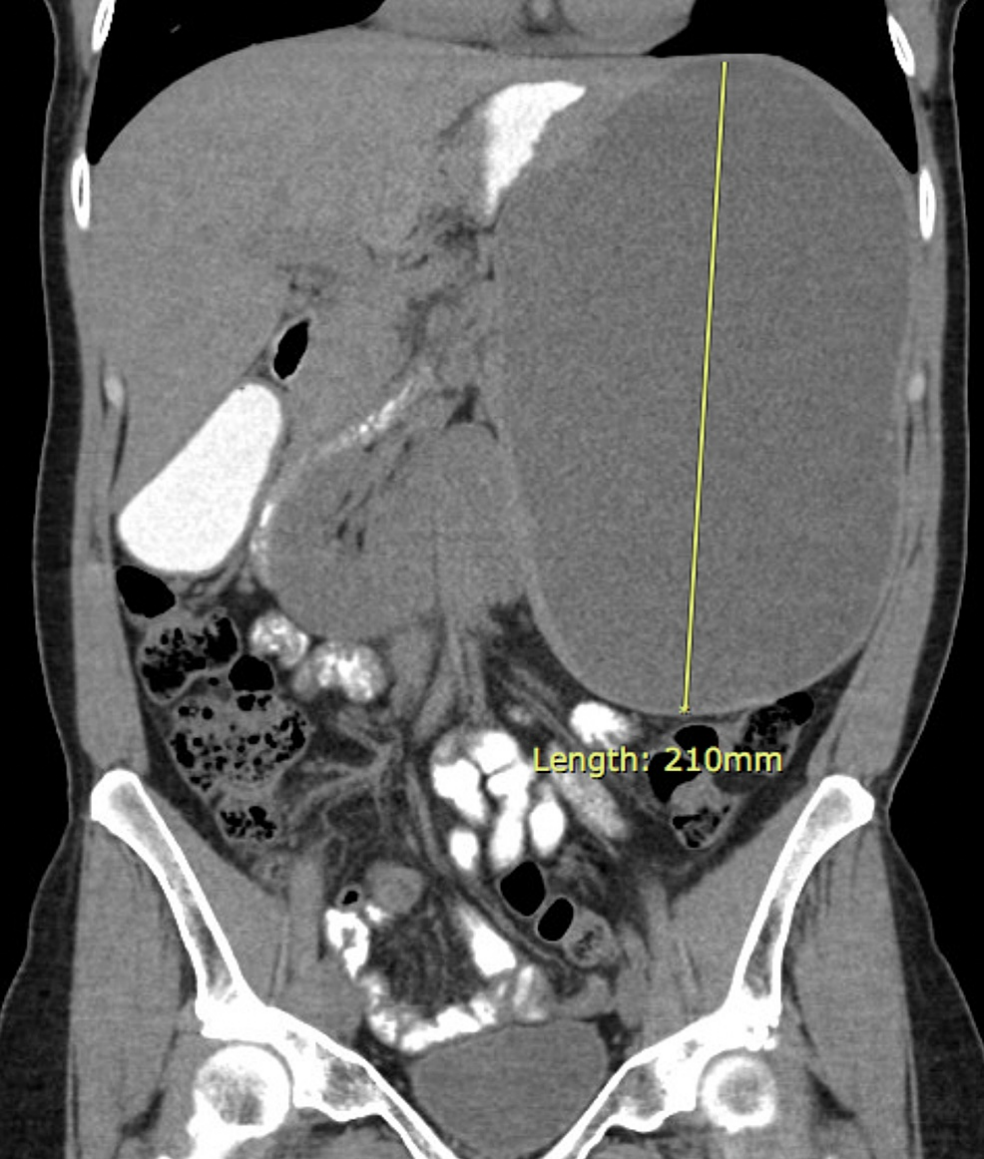
Abdominal CT scan demonstrating the splenic cyst

**Figure 2:**
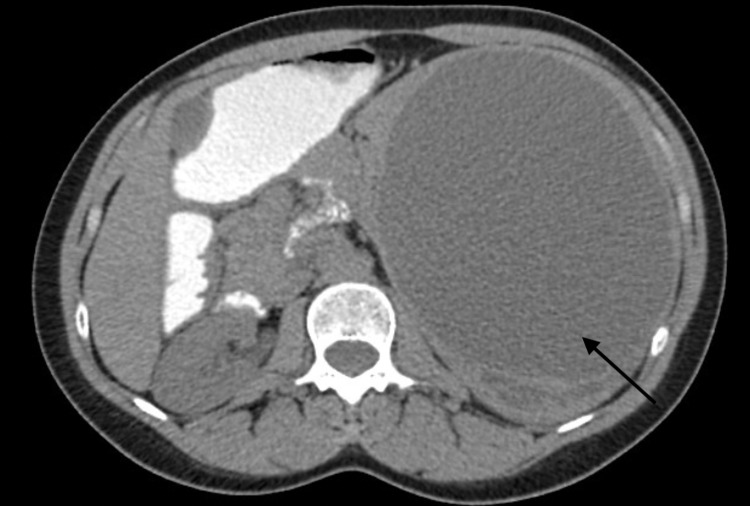
Abdominal CT scan illustrating the cystic mass with several posterior loculations

Further imaging was obtained (Figures 1-2). CT without contrast of the abdomen and pelvis showed an intrasplenic large cystic homogenous mass measuring 13 x 15 x 21 cm. MRI with and without contrast was recommended by the radiologist for further evaluation of the mass to provide greater resolution of the loculations and for a more definitive diagnosis. It was also recommended because the CT scan was conducted without IV contrast. The MRI showed an enlarged spleen that contained a 21.1 x 16.5 x 13.1 cm T2/T1 hyperintense non-enhancing lesion with a large dominant locule and several small locules posteriorly with non-enhancing septations. The radiologist suggested a pseudocyst with proteinaceous or hemorrhagic contents, less likely to be a primary epithelial cyst, given the multiple loculations, or a hydatid cyst, given the homogeneity.

Due to the extensive size of the mass, with the possible compression of the stomach causing the early satiety, a thorough discussion of the risks versus benefits and potential complications was discussed with the patient. The decision was made to schedule an elective laparoscopic-assisted splenectomy vs. open splenectomy. The patient received the appropriate vaccinations against encapsulated organisms.

The patient was placed in a supine position on the operating table. A 5 cm longitudinal periumbilical incision was made and a GelPOINT port (Applied Medical Resources Corporation, Rancho Santa Margarita, California) was inserted with four ports in situ. The abdominal cavity was insufflated to a pressure up to 12 mmHg. After evaluating the large spleen, the splenocolic ligament was mobilized. Once the inferior edge of this plane was dissected free, the lesser sac was explored and the short gastric vessels ligated with a LigaSure sealer (Medtronic plc, Dublin, Ireland). The hilar vessels were carefully dissected. The spleen was very large, and access to the hilum of the spleen was limited. A decision was then made to convert to open splenectomy. Bleeding was encountered from the splenic capsule, which was located close to the hilum and was controlled with a lap pad pressure; the periumbilical incision was extended to the epigastric region. Due to the enlargement of the spleen, the procedure required Ochsner trocar decompression with a purse-string suture that was connected to suction for decompression to prevent spillage. The decompressed fluid consisted of two liters of brownish fluid that was sent for culture, cytology, and gram staining. No occurrences of spillage were detected. The splenic artery and vein that was previously dissected during the laparoscopic approach was doubly ligated and divided with a LigaSure, which allowed for further mobilization of the splenic hilum and attachments. The spleen was removed and sent for routine pathology. The patient was awakened, extubated, and transported to the post-anesthesia care unit (PACU) in stable condition. Recovery was uneventful, and the patient didn't experience any complications postoperatively.

**Figure 3:**
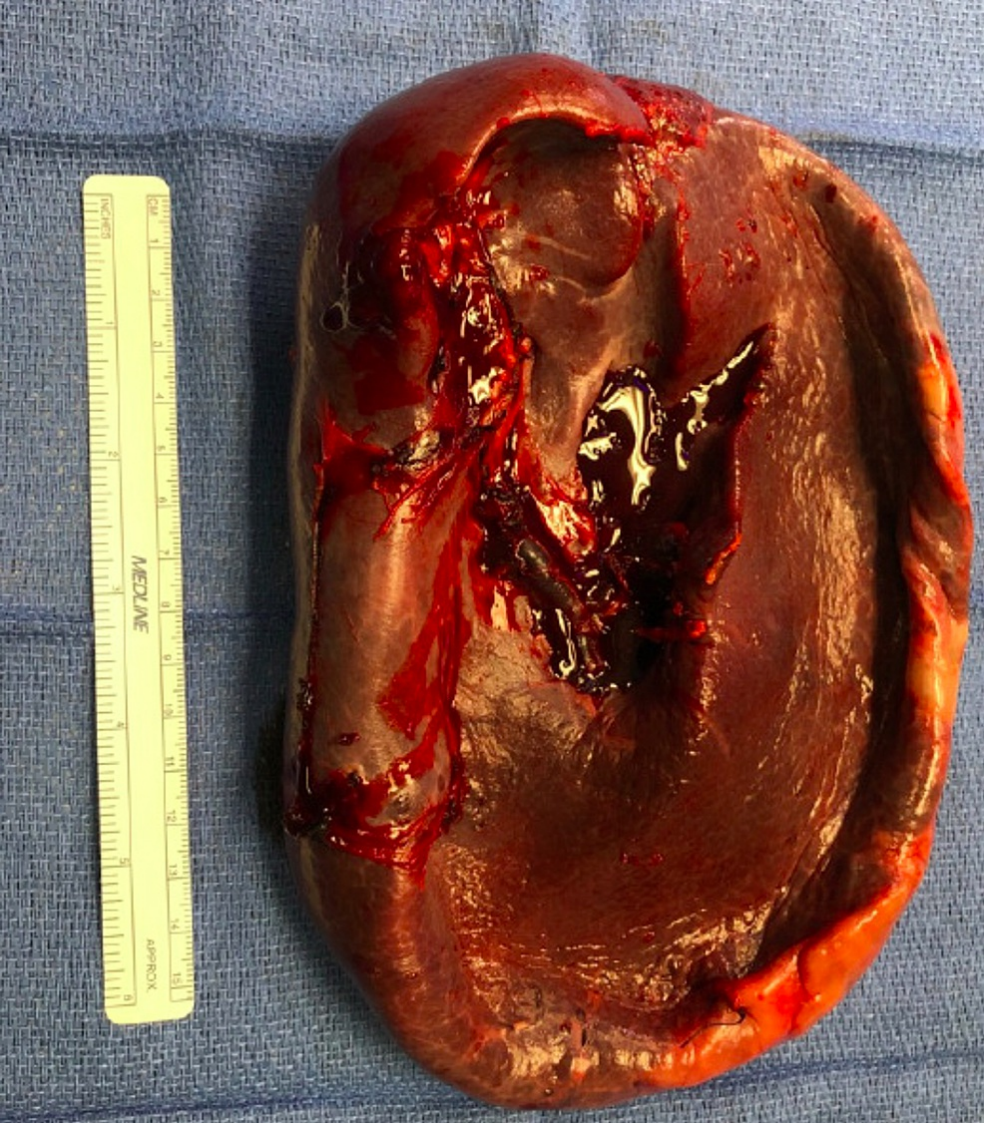
Gross pathology of the enlarged spleen

**Figure 4:**
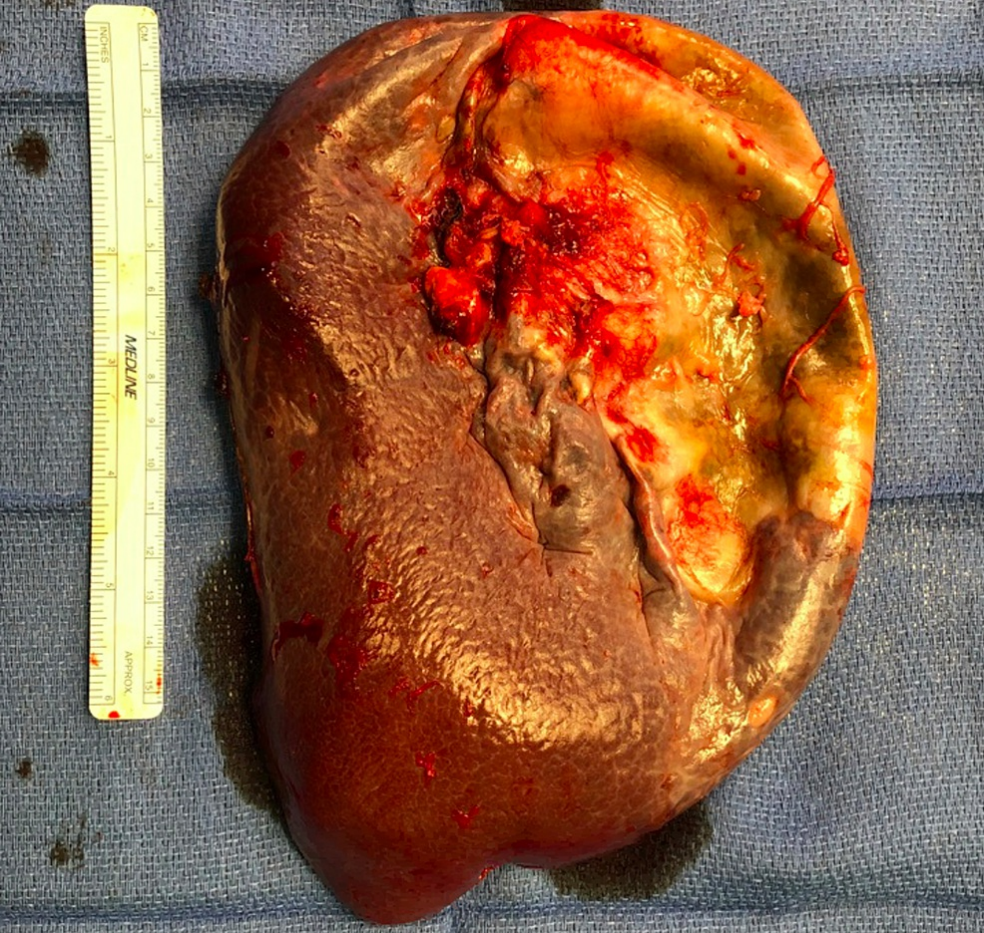
Gross pathology of the spleen with the decompressed splenic cyst

The pathology report showed an enlarged spleen that weighed 454 grams and measured 19 x 12 x 4 cm with a thickened capsule (Figure 3). After decompressing the cyst, gross examination measured approximately 13 cm in the largest dimension with brown fluid (Figure 4). The cyst appears unilocular with trabecular fibrous tissue lining it. Microscopic findings revealed red pulp congestion and follicular lymphoid hyperplasia of the white pulp; there was no morphological evidence of involvement of malignant lymphoma. The adjacent, large cystic structure had a fibrous capsule lined partially by stratified squamous epithelium, with the remainder of the cyst lined by macrophages and hemorrhagic material. No atypia was noted. Focal cholesterol cleft formation was seen within the squamous debris. These histological findings are most compatible with a splenic epithelial cyst with some serosal adhesion throughout the external aspect of the cyst wall.

## Discussion

This case represents a large splenic epidermoid cyst occupying the left hypochondrium area. In 1958, Martin et al. classified splenic cysts as follows: epidermoid cysts are considered Type I non-parasitic, which are further classified as congenital and neoplastic cysts; congenital cysts include epidermoid, dermoid, and endodermoid, which are histologically identified by the inner epithelial lining of the stratified squamous epithelium [3]. The origin of primary cysts remains unclear, yet they are thought to have developed from the entrapment of peritoneal mesothelial cells in the splenic parenchyma during embryogenesis in utero [4]. Neoplastic cysts of the spleen consist of hemangiomas, the most common of neoplastic origin, and lymphangiomas. The other form of Type I primary cysts is parasitic, which is caused in areas that are endemic to the tapeworm Echinococcosis granulosus.

Surgical treatment for splenic cysts remains controversial; however, selecting the most optimal surgical approach is determined by the location, depth, and size of the cyst as well as weighing out the risks versus benefits for the patient. Indications for a total splenectomy include multiple cysts, very large-sized (>5 cm), the location of the cyst - specifically in the hilum of the spleen, intrasplenic cysts that are covered completely by the splenic parenchyma, and uncontrollable intraoperative bleeding [5-7]. Our patient’s CT scan and MRI findings confirmed a very large intrasplenic cystic mass measuring 16 x 12.5 cm; therefore, a total splenectomy was conducted due to the possibility of rupturing or bleeding. Initially, the procedure was conducted as a minimally invasive procedure, but the splenic cyst was too large, with concern that the cyst may cause spillage or rupture. Consequently, the decision was made to convert to an open procedure to facilitate drainage in a controlled fashion and prevent spillage of the cystic contents into the abdominal cavity. Considerations were made after the conclusion of the procedure; it was thought that percutaneous drainage prior to the operation could have prevented the conversion to open splenectomy.

Percutaneous drainage in the treatment of splenic cysts is debatable. Some authors believe it is a safe, cost-effective, and efficacious modality of treatment that ensures early convalescence. Others believe that this method has a high incidence of recurrence [5-6]. The advantage of percutaneous drainage can be used as a preoperative method to decrease the size of the large cysts, to allow for easier access to vital structures, and for maneuvering techniques to extract the spleen [7]. Another case report mentions the advantages of percutaneous drainage as a bridge to laparoscopic surgery to plan for the best treatment for the specific cyst. It also stated that decapsulation is not recommended due to the risk of recurrence; therefore, they proposed that partial or total splenectomy is a definitive treatment [8]. This reported case confirmed that their patient underwent percutaneous drainage prior to laparoscopic surgery for total splenectomy and had a complete recovery.

For many years the preferred method was open total splenectomy; however, nowadays, a spleen-preserving minimally invasive approach is beneficial because it reduces the risk of uncontrolled postoperative infections, minimizes the post-operative healing time with the return to full activity, and is cosmetically beneficial. On the other hand, total splenectomies are recommended because it prevents recurrence, which is attributed to hidden tiny cysts that remained at the time of the procedure. It also prevents infection, rupture, or hemorrhage that potentially leads to serious complications.

There are other approaches of operative management for the excision of nonparasitic splenic cysts, which include marsupialization and fenestration (partial cystectomy, unroofing, deroofing). To summarize the extensive literature reviewed, treatment options for primary splenic cysts are focused on minimally invasive surgical approaches with the preservation of the spleen.

## Conclusions

Total splenectomy was indicated for the treatment of our patient with a splenic cyst of size larger than 5 cm and location close to the hilum of the spleen. A laparoscopic approach was first attempted but due to its size, there was concern that the cyst may rupture and spill into the abdominal cavity, causing further complications. Treatment with percutaneous drainage before total splenectomy may be considered in patients with large cysts. This method has been shown to be safe in previous case reports and allows decompression of the cysts, giving better visibility and easier access for total splenectomy; however, recent studies have focused their attention on minimally invasive approaches and spleen-preserving techniques.

## References

[1] Marjanović ZO, Djordjević IM (2008). Epidermoid splenic cysts in children and adolescents [Article in Serbian]. Acta Chir Iugosl.

[2] Rana AP, Kaur M, Singh P, Malhotra S, Kuka AS (2014). Splenic epidermoid cyst - a rare entity. J Clin Diagn Res.

[3] Fahel E, Amarala PC, Filho EM (2000). Videolaparoscopic approach of the splenic cyst: a case report. JSLS.

[4] Algino SE, Sorrentino S, Luyimbazi DT, Grider DJ (2019). Epidermoid cysts in a wandering spleen: an unusual enigma. Case Rep Surg.

[5] Karfis EA, Roustanis E, Tsimoyiannis EC (2009). Surgical management of nonparasitic splenic cysts. JSLS.

[6] Smith ST, Scott DJ, Burdick JS, Rege RV, Jones DB (2001). Laparoscopic marsupialization and hemisplenectomy for splenic cysts. J Laparoendosc Adv Surg Tech A.

[7] Macheras A, Misiakos EP, Liakakos T, Mpistarakis D, Fotiadis C, Karatzas G (2005). Non-parasitic splenic cysts: a report of three cases. World J Gastroenterol.

[8] Morandi E, Castoldi M, Merlini DA, Vignati G, Milanesi M (2012). Is there a role of percutaneous drainage in non-parasitic splenic cysts? Case report. G Chir.

